# EXTERNAL VALIDATION OF THE DIAREM SCORE AS REMISSION PREDICTOR OF DIABETES MELLITUS TYPE 2 IN OBESE PATIENTS UNDERGOING ROUX-EN-Y GASTRIC BYPASS

**DOI:** 10.1590/S0102-6720201500S100007

**Published:** 2015-12

**Authors:** José SAMPAIO-NETO, Luís Sérgio NASSIF, Alcides José BRANCO-FILHO, Luciana Alves BOLFARINI, Luiara Stefanelo LORO, Mayara Prudêncio de SOUZA, Thais BIANCO

**Affiliations:** Pontifical Catholic University of Paraná, Curitiba, PR, Brazil

**Keywords:** Bariatric surgery, Gastric Bypass, DiaRem score, Diabetes Mellitus, type 2

## Abstract

***Background*:**

DiaRem score consists in preoperative model for predicting remission of type 2 diabetes mellitus in obese patients who underwent gastric bypass.

***Aim*:**

To evaluate the applicability of DiaRem comparing the scores obtained preoperatively with remission of T2DM after surgery.

***Method*:**

Preoperative parameters such as age, use of insulin, oral hypoglycemic agents and glycated hemoglobin, were retrospectively evaluated in diabetic patients undergoing gastric bypass during the period between July 2012 to July 2013. Through these data the DiaRem score were applied. The results of fasting blood glucose and glycated hemoglobin were requested prospectively.

***Results*:**

Were selected 70 patients; the remission of T2DM after surgery was found in 42 (60%) and no remission in 28 (40%). Checking the final score, it was observed that: from 0 to 2 points, 94.1% of patients remitted completely; between 3 and 7 had remission in 68.9%, of which 42.8% complete; from 8 to 12, 57.1% achieved complete remission; between 13 to 17, 87.5% did not achieve remission and was not seen this complete remission group; between 18 to 22, 88.9% were not remitted.

***Conclusion*:**

The DiaRem score showed appropriate tool to assess remission of T2DM in obese patients who will undergo gastric bypass.

## Abstract (pt)

***Racional*:**

Escore DiaRem consiste em modelo pré-operatório de predição de remissão de diabete melito tipo 2 (DM2) em pacientes obesos que serão submetidos exclusivamente ao bypass gástrico.

**Objetivo:**

: Avaliar a aplicabilidade desse escore comparando a pontuação obtida pré-operatoriamente com a remissão de DM2 após realização da operação.

**Método:**

: Foram avaliados retrospectivamente, dados clínicos e laboratoriais pré-operatórios necessários para aplicar o escore (idade, uso de insulinoterapia, uso de hipoglicemiantes orais e hemoglobina glicada) de pacientes diabéticos submetidos à gastroplastia laparotômica do tipo bypass, e após um a dois anos de acompanhamento pós-operatório, verificou-se a remissão ou não do DM2.

***Resultados*:**

Selecionou-se 70 pacientes; a remissão completa foi encontrada em 35 (50%), parcial em sete (10%) e não houve remissão em 28 (40%). Verificando a pontuação final, foi visto que as expectativas lançadas pelo escore DiaRem foram atingidas, pois o grupo de pacientes que alcançou a remissão foi o mesmo que obteve as menores pontuações.

**Conclusão:**

: O escore DiaRem mostrou-se ferramenta apropriada para avaliar remissão de DM2 em obesos que serão submetidos ao bypass gástrico.

## INTRODUCTION

Diabetes mellitus type 2 (DM2) is heterogeneous group of diseases that share common elements, the most important being the hyperglycemia^5^^,^ 8. It is known as chronic disease caused by multiple factors and, among the risk factors, obesity is the main known environmental factor^11^^,^ 18. Excess body fat results in imbalance of resistin and adiponectin hormones, which are produced by adipose tissue^14^. The first reduces the ability of insulin to properly metabolize glucose; already adiponectin promotes opposite effect by facilitating the action of insulin. In obese people increases the production of resistin and adiponectin declines, resulting in insulin resistance and favoring the appearance of DM2^14^.

The current medical approach to diabetic obese patients implies to recommend weight loss through medical treatment^4^^,^ 13. However, with the advancement of medicine and the new techniques of bariatric surgery used for the treatment of morbid obesity was observed relationship between these operations and improved glucose levels in patients undergoing operation^14^. Among the existing procedures, gastric bypass is one of the techniques shown to improve the DM2^14^.

Despite encouraging results with surgery, it is known that the post-surgical not always has expectation to disease remission^7^^,^ 9. Thus, in order to predict DM2 remission in patients who underwent only gastric bypass technique in Y-de-Roux was formulated in January 2014 DiaRem score^16^. It is based on age, glycated hemoglobin, oral hypoglycemic drugs and insulin. For each criterion there is a specific score that after being added, estimates the probability of DM2 remission after the procedure^12^^,^ 16.

Knowing the importance of this study that in the future will enable base surgical indications, not only using body mass index criteria but also the individual differences inherent in this disease, this study aimed to evaluate the applicability of the score DiaRem comparing the score obtained preoperatively with remission of DM2 after the completion of the gastric bypass, being this communication the first foreign population to validate it.

## METHOD

The Ethics Committee of the Pontifical Catholic University of Paraná approved this study and individuals selected for the sample were included in the selection criteria of the Brazilian Society for Bariatric and Metabolic Surgery^11^^,^ 12.

It is retrospective analytical observational study by analyzing electronic medical records for Bariatric Surgery Clinic patients of the Brotherhood of Santa Casa de Misericordia Hospital in the city of Curitiba, Paraná, Brazil.

The sample selection was made from electronic medical records. Initially they found 220 obese diabetic patients submitted to different techniques of bariatric surgery. Of these, 94 underwent Roux-en-Y gastric bypass between July 2012 and July 2013 had at least 12 months of postoperative follow-up and were operated and evaluated before and after surgery by the same surgical team.

Patients who did not meet established criteria^10^ for diagnosis of DM2 were excluded, also those who underwent other surgical techniques other than Roux-en-Y gastric bypass, those with less than 12 months of surgical procedure and those whose electronic medical records had missing data for the realization of the project.

The data required for this project comprised the mandatory protocol of preoperative assessment and postoperative follow-up applied in service. All postoperative laboratory tests were performed in the same laboratory.

Follow-up was performed at 3, 6, 9, 12, 18 and 24 months after the surgery. They were also treated at the aforementioned clinic if symptoms develop between the usual follow-up interval.

Preoperative data collected included age, gender, use of oral hypoglycemic drugs, use of insulin, fasting glucose and glycated hemoglobin.

Postoperative data included use of oral hypoglycemic drugs, use of insulin, fasting glucose and glycated hemoglobin.

### Score assessment DiaRem

The score of the patients was obtained according to the DiaRem score, which uses four preoperative parameters to allow for the remission of DM2 after performing gastric bypass, namely: 1) glycated hemoglobin; 2) age; 3) use of insulin; and 4) use of oral hypoglycemic agents. The score is punctuated 0-22, divided into items and sub-items, each receiving certain score (Table 1).

Patients were stratified to five score subgroups: 0-2; 3-7; 8-12; 13-17 and 18-22, as proposed by Still et al 9. The reference DM2, in turn, was divided into complete, partial or non-existent. Groups of 0-2; 3-7; 8-12; 13-17 and 18-22 have been inserted into any of three reference variables (complete, partial or no).

**TABLE 1:** - Punctuation model based on DiaRem score

**Parameters**	**Punctuation**
Age (years)	
< 40	0
40-49	1
50-59	2
>= 60	3
HbA1c (%)	
< 6,5	0
6,5 - 6,9	2
7,0- 8,9	4
>9,0	6
Use of oral hypoglycemic	
Only metformin	0
Sulfonylureas and other insulin sensitizing agents except metformin	3
Insulin treatment	
No	0
Yes	10

Still *et al.* Preoperative prediction of type 2 diabetes remission after Roux-en-Y gastric bypass surgery: a retrospective cohort study. Lancet Diabetes Endocrinol. 2014; 2: 38-45.

According to criteria of the American Diabetes Association is considered remission of type 2 diabetes: 1) full: fasting glucose below 100 mg/dl and glycated hemoglobin below 6.0% without using hypoglycemic; 2) partial: fasting glucose between 100-125 mg/dl and glycated hemoglobin between 6.0-6.5%, without the use of hypoglycemic; 3) zero: when the above criteria are not met. To analyze the remission of selected patients, this study sought fasting blood glucose levels, glycated hemoglobin and use of hypoglycemic after 12 months post-surgery.

### Data analysis

Data were organized into Excel(r) spreadsheet, analyzed using the software Statistica v.8.0^(r)^ and the results of quantitative variables were described as mean, median, minimum and maximum values, and standard deviations. Qualitative variables were expressed as frequencies and percentages. In relation to qualitative variables comparisons were made using Fisher's exact test. P values ​​<0.05 were considered statistically significant. Data were also analyzed with the computer program SPSS v.20.

## RESULTS

Of the 94 selected patients, 24 (25.5%) were excluded for not meeting the established selection criteria. Therefore, the final sample consisted of 70 subjects (n=70), representing 74.5% of previously evaluated. Complete remission of DM2 was found in 35 (50%), partial in seven (10%), totaling 42 patients who achieved remission (60%); there was no remission in 28 (40%).

The final sample, 64 participants (91.4%) were women and six (8.6%) men; the preoperative mean age was 47.9±9.9 years. Analyzing the age group separately, it was shown that the average of the participants who achieved remission was 44.9 years and 52.5 years in those who had not, with a standard deviation of 10.5 and 7.1 respectively (p<0.001). In this sample, the age of 50 years was significantly related to non remission (p=0.028).

The analysis of the preoperative data in relation to the hypoglycemic therapies, it was observed that 25 subjects (35.7%) were taking metformin alone; 17 (17.1%) metformin associated with other oral hypoglycemic agents; five (7.1%) combined with other sulfonylurea insulin sensitizers except metformin; and 18 (25.7%) insulin. With regard to preoperative blood biochemistry, mean fasting blood glucose was 130.7±53.1 mg/dl and glycated hemoglobin of 7.6±1.8%, being above the recommended levels for diabetics.

**TABLE 2:** - Retrospective analysis of DiaRem score variables according to remission of type 2 diabetes after gastric bypass

**Data**	**Metformin alone**	**Combined use of the sulfonylurea insulin sensitizers**	**Insulin therapy**	**Fasting glucose**	**Glycated hemoglobin**
Pre-operative					
Remission DM2					
Yes (n=42)	24 (57.1%)	3 (7.1%)	2 (4.7%)	121.6±51,1	7.2±1.8
No (n=28)	6 (21.4%)	2 (7.1%)	16 (57.1%)	144.2±54.1	8.1±1.6
Post-operative					
Remission DM2					
Yes (n=42)	0 (0,0%)	0 (0.0%)	0 (0.0%)	89.0±8,8	5.5±0.5
No (n=28)	17 (60.7%)	5 (17.8%)	4 (14.2%)	125.1±45.5	6.9±1.9

Among patients who did not achieve remission, the use of preoperative insulin was factor statistically significant compared to those who did not use it and achieved remission (p<0.001). Moreover, the use of metformin alone was positively associated with postoperative remission (p=0.017). Comparison the pre- and postoperative showed up fewer hypoglycemic drug users and decrease in fasting glucose levels and glycated hemoglobin in both patient groups (Table 2).

### DiaRem score applicability 

In the final participants sample classification in accordance with the DiaRem score intervals there was 17 subjects (24.3%) with 0 to 2 points; 29 (41.4%), 3 to 7 points; 7 (10%), 8 to 12; 8 (11.4%), 13 to 17; and 9 (12.9%) scored between 18 and 22.

To evaluate the applicability of DiaRem score were compared the scores obtained preoperatively with the remission of DM2 after performing the gastric bypass (Table 3). Note that, among those who showed complete remission, the highest percentage was found in ranges 0 to 2 and 3 to 7, and no obtained value equal to or above 13 points. Among those who had no remission, although it can be seen higher division between intervals, was composed almost for all (88.9%) allocated in the range of 18 to 22 (Table 3).

**TABLE 3:** - Distribution of the sample according to score and remission of DM2

**Score**	**Remission**	**Total**		
	**Complete**	**Partial**	**Without remission**	
0 a 2	16 (45.7%)	0 (0.0%)	1 (3.6%)	17 (24.3%)
3 a 7	15 (42.8%)	5 (71.4%)	9 (32.1%)	29 (41.4%)
8 a 12	4 (11.4%)	0 (0.0%)	3 (10.8%)	7 (10%)
13 a 17	0 (0.0%)	1 (14.3%)	7 (25%)	8 (11.4%)
18 a 22	0 (0.0%)	1 (14.3%)	8 (28.6%)	9 (12.9%)
Total	35 (50%)	7 (10%)	28 (40%)	70 (100%)

 The comparison between the number of participants who acquired remission and scored from 0 to 2 versus those who did not and achieve a score greater than or equal to 18 was statistically significant (p<0.001), indicating that the shown distribution cannot be assigned to chance.

**FIGURE 1:**
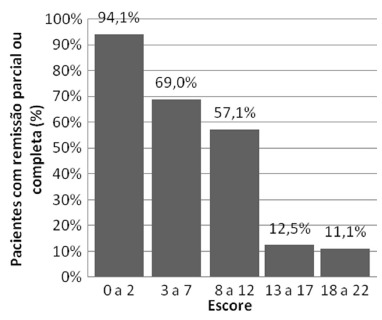
- DiaRem score intervals and remission of DM2

Figure 1 shows the division of the score related to the reference. It is observed that in minute intervals, 0-2 and 3-7, the percentage of patients with remission after operation consisted of 94.12%and 68.97%, respectively. On maximum interval there was one remission (11.11%).

To determine a cut point for the score that is associated with the remission (partial or complete) a ROC curve was fitted. The area under the curve is equal to 0.841, significantly greater than 0.5 (p<0.001). This result indicates well discrimination between the score having remission (partial or complete) and having no remission. The optimal cutoff point (better sensitivity and better specificity) is denoted by the curve 7. The sensitivity of this cutting point, ie, the probability of scoring below 7 when there is full or partial remission is 83.3% (95% CI: 72.1% to 94.6%). Already, the positive predictive value of the model, when the score is less than 7, is 81.4% (95% CI: 69.8% to 93.0%); for this calculation the prevalence of cases of remission was estimated from the study sample.

## DISCUSSION

The specific mechanisms that lead to the positive effects of bariatric surgery on diabetes are still in debate^3^. It is understood that the resistance to insulin action - a major component of this disease - decrease in weight loss. However, the difference observed between disabsorptive and restrictive procedures, show that weight loss is not the only factor involved in the control of blood glucose levels without hypoglycemic medications after the operation. Indeed, gastric bypass promotes insulin secretion due to hormonal changes, particularly in levels of incretins and glucagon-like peptide-1 (GLP-1)^6^. However, this stimulatory action of insulin production requires the presence and proper functioning of pancreatic beta cells^6^.

Clinical studies with groups of obese containing individuals with class I obesity to severe obesity, and who had remission of type 2 diabetes after gastric bypass, have demonstrated similar preoperative clinical profile^1^^,^ 2. Young patients with lower diabetes duration and absence of preoperative insulin therapy, showed that these data are significant predictors of remission, regardless of the percentage of weight loss. It was clearly demonstrated their importance as clinical indicators of beta cell function and its correlation with improved post-operative glycemic response^1^^,^ 2 ^,^ 7.

As discussed previously, the score DiaRem created by Still et al consists of three clinical and laboratory variables as DM2^16^remission predictors. A retrospective analysis of medical records of diabetic patients who underwent gastric bypass, these authors reported that 87% of patients were scored 0-2 points; 66%, 3 to 7; 32%, 8 to 12; 16% ,13 to 17; and 5%, 18 to 22 points in 12 months remission after the procedure^16^. However, until the time of the present study, the score was not applied in any other population besides the one studied by those authors^16^.

Thus, the main focus of this study was to evaluate the applicability of DiaRem score by comparing the scores obtained preoperatively with the remission of type 2 diabetes after submission of diabetic obese patients to bypass. This research has found similar results to the original article, for both analysis showed similarities in the distribution percentage in DiaRem intervals among participants who achieved remission, confirming that low scores predict high probability of remission of type 2 diabetes, while high scores showed low expectation to remission.

The preoperative variables considered, such as age, use of metformin and insulin were significantly associated with remission or not remission of DM2 in this study and corroborate the relevance of the analysis of these factors in diabetic individuals who carry out gastric bypass. The duration of diabetes could not be analyzed in this sample due to the difficulty in dating the onset of the disease by patients. However, it appears that patients over 50 had a greater duration of the disease and therefore had lower response to incretinic effects after operation. Similarly, younger individuals and/or who did not use insulin, so probably with shorter disease and injury to pancreatic beta cells, were those who had better glycemic control.

Some limitations of this study should be mentioned. Firstly, the relatively small sample size as well as the retrospective data collection, restricted more complex analysis. Secondly, the follow-up period was short, therefore it cannot be assured the predictive score to long-term follow-up. Another point to note is that although the BMI is a criterion for surgical indication, there is no conclusive evidence showing that certain preoperative values ​​are related to remission of type 2 diabetes after gastric bypass, and so was not selected as a predictor for this study^3^^,^ 10 ^,^ 15 ^-^ 17 ^,^ 19. Despite the findings, the statistical power obtained with the results of this study infers the effectiveness and reliability of using DiaRem score as a predictor of type 2 diabetes remission in patients who carry out gastric bypass, mainly allocated for individuals in the lowest score range. Furthermore, the score proved to be simple, requiring no special equipment and providing potential applicability in clinical practice.

## CONCLUSION

The DiaRem score proved to be valid tool to assess type 2 diabetes remission in obese patients undergoing gastric bypass.
